# Feasibility of the cardiac output response to stress test in suspected heart failure patients

**DOI:** 10.1093/fampra/cmab184

**Published:** 2022-01-27

**Authors:** Sarah J Charman, Nduka C Okwose, Clare J Taylor, Kristian Bailey, Ahmet Fuat, Arsen Ristic, Jonathan Mant, Christi Deaton, Petar M Seferovic, Andrew J S Coats, F D Richard Hobbs, Guy A MacGowan, Djordje G Jakovljevic

**Affiliations:** Translational and Clinical Research Institute, Faculty of Medical Sciences, Newcastle University, Newcastle upon Tyne, United Kingdom; Newcastle upon Tyne Hospitals NHS Foundation Trust, Newcastle upon Tyne, United Kingdom; Translational and Clinical Research Institute, Faculty of Medical Sciences, Newcastle University, Newcastle upon Tyne, United Kingdom; Newcastle upon Tyne Hospitals NHS Foundation Trust, Newcastle upon Tyne, United Kingdom; Research Centre (CSELS), Institute for Health and Wellbeing, Faculty of Health and Life Sciences, Coventry University, and University Hospitals Coventry and Warwickshire NHS Trust, Coventry, United Kingdom; Nuffield Department of Primary Health Care Sciences, University of Oxford, Oxford, United Kingdom; Newcastle upon Tyne Hospitals NHS Foundation Trust, Newcastle upon Tyne, United Kingdom; Darlington Memorial Hospital, County Durham and Darlington NHS Foundation Trust & School of Medicine, Pharmacy and Health, Durham University, Durham, United Kingdom; Department of Cardiology, Faculty of Medicine, University of Belgrade, Clinical Centre of Serbia, Belgrade, Serbia; Primary Care Unit, Department of Public Health and Primary Care, University of Cambridge, Cambridge, United Kingdom; Primary Care Unit, Department of Public Health and Primary Care, University of Cambridge, Cambridge, United Kingdom; Department of Cardiology, Faculty of Medicine, University of Belgrade, Clinical Centre of Serbia, Belgrade, Serbia; Department of Medical Sciences, Centre for Clinical and Basic Research, IRCCS San Raffaele Pisana, Rome, Italy; Nuffield Department of Primary Health Care Sciences, University of Oxford, Oxford, United Kingdom; Newcastle upon Tyne Hospitals NHS Foundation Trust, Newcastle upon Tyne, United Kingdom; Biosciences Institute, Newcastle University, Newcastle upon Tyne, United Kingdom; Translational and Clinical Research Institute, Faculty of Medical Sciences, Newcastle University, Newcastle upon Tyne, United Kingdom; Newcastle upon Tyne Hospitals NHS Foundation Trust, Newcastle upon Tyne, United Kingdom; Research Centre (CSELS), Institute for Health and Wellbeing, Faculty of Health and Life Sciences, Coventry University, and University Hospitals Coventry and Warwickshire NHS Trust, Coventry, United Kingdom

**Keywords:** cardiac output, feasibility, general practice, heart failure, primary care

## Abstract

**Background:**

Diagnostic tools available to support general practitioners diagnose heart failure (HF) are limited.

**Objectives:**

(i) Determine the feasibility of the novel cardiac output response to stress (CORS) test in suspected HF patients, and (ii) Identify differences in the CORS results between (a) confirmed HF patients from non-HF patients, and (b) HF reduced (HFrEF) vs HF preserved (HFpEF) ejection fraction.

**Methods:**

Single centre, prospective, observational, feasibility study. Consecutive patients with suspected HF (*N* = 105; mean age: 72 ± 10 years) were recruited from specialized HF diagnostic clinics in secondary care. The consultant cardiologist confirmed or refuted a HF diagnosis. The patient completed the CORS but the researcher administering the test was blinded from the diagnosis. The CORS assessed cardiac function (stroke volume index, SVI) noninvasively using the bioreactance technology at rest-supine, challenge-standing, and stress-step exercise phases.

**Results:**

A total of 38 patients were newly diagnosed with HF (HFrEF, *n* = 21) with 79% being able to complete all phases of the CORS (91% of non-HF patients). A 17% lower SVI was found in HF compared with non-HF patients at rest-supine (43 ± 15 vs 51 ± 16 mL/beat/m^2^, *P* = 0.02) and stress-step exercise phase (49 ± 16 vs 58 ± 17 mL/beat/m^2^, *P* = 0.02). HFrEF patients demonstrated a lower SVI at rest (39 ± 15 vs 48 ± 13 mL/beat/m^2^, *P* = 0.02) and challenge-standing phase (34 ± 9 vs 42 ± 12 mL/beat/m^2^, *P* = 0.03) than HFpEF patients.

**Conclusion:**

The CORS is feasible and patients with HF responded differently to non-HF, and HFrEF from HFpEF. These findings provide further evidence for the potential use of the CORS to improve HF diagnostic and referral accuracy in primary care.

**Key messages**

- Suspected heart failure patients were able to complete the CORS test.
- Different results were found between heart failure and nonheart failure patients.
- Differences were found between ejection fraction groups.

## Background

Heart failure (HF) is a complex clinical syndrome associated with impaired heart function at rest and/or during exertion.^1,2^ HF is recognized as a global health and economic burden,^3,4^ affecting nearly 1 million people in the United Kingdom.^1,2,5^ HF is a serious chronic disease with poor prognosis, poor quality of life and high healthcare costs.^2,6^ Five-year mortality rates of nearly 42% are mostly due to myocardial infarction and worsening HF.^7^ The prevalence of HF and consequential hospitalization are expected to rise due to our ageing population^8^ affecting ≥10% in individuals >70 years of age.^1^

Typical signs and symptoms of HF include breathlessness, ankle swelling, and fatigue,^1^ which are very common and nonspecific. This makes diagnosis challenging due to difficulties in differentiating between HF and other conditions associated with ageing, obesity, or lung disease.^1,9^ In addition, multimorbidity and polypharmacy can complicate the diagnosis further.^10^ Early and precise diagnosis of HF is therefore essential in order to apply appropriate treatment, which can improve patient’s morbidity, quality of life, and mortality^11^ as well as reducing NHS burden of HF.^1,2,12^

Primary care represents an entry point in the clinical care pathway for patients presenting with HF signs and symptoms and general practitioners (GPs) play a key role in identifying HF.^11,13,14^ Currently, the diagnostic tools available in primary care to help diagnose HF include a full medical history, signs and symptoms, electrocardiography, and serum natriuretic peptides test.^1^ However, electrocardiography seems to be an inadequate screening tool for patients with suspected HF as described by a systematic meta-analysis.^15^ The serum natriuretic peptides blood test is useful as a “rule out” test but results in a high number of false positives.^11,16–18^ Furthermore, this diagnostic test continues to be underutilized by GPs in primary care.^19^ In practice, all these assessments are used to establish a HF diagnosis, which also therefore requires some specialist clinical interpretation. It is therefore not surprising that current diagnostic practice of HF in primary care results in high number of inaccurate and expensive referrals to secondary care.^15,20–22^ Diagnostic uncertainty and delayed diagnosis of HF can lead to incorrect treatment, impaired disease progression and increased hospitalization.^11^

**Fig. 1.**
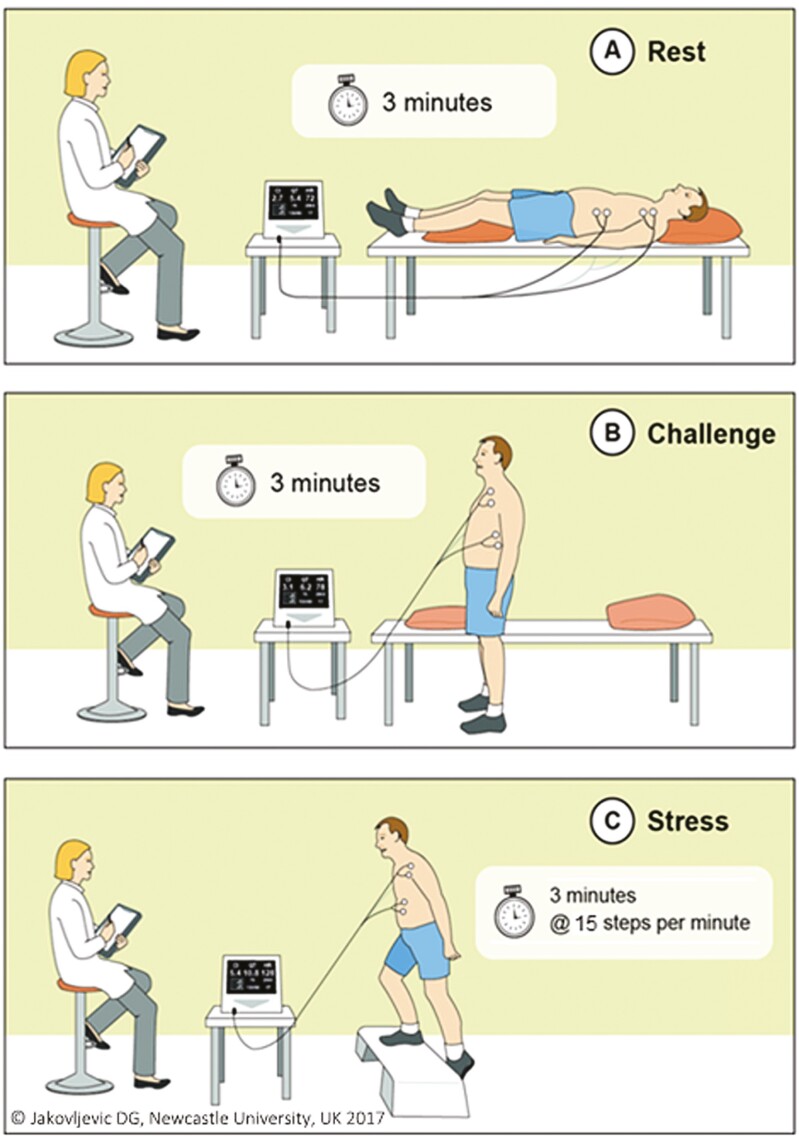
Timeline of measurements. T1–T4, time points of measurement; LAS, lateral ankle sprain.

The COVID-19 pandemic has further reinforced that new strategies to achieve timely diagnosis due to patients avoiding urgent care for HF signs and symptoms^23,24^ in primary care should be a priority for future research and policy as most recently suggested.^25^ To help mitigate this challenge, we have developed and recently confirmed acceptable reproducibility of a novel, easy-to-administer, noninvasive cardiac output response to stress (CORS) test^26^ (Fig. 1). Our recent qualitative work with healthcare professionals in primary and secondary care highlighted a high demand for an additional diagnostic tool within primary care to aid diagnostic and referral accuracy.^27^ We hypothesis that the CORS test may enable a new clinical pathway—once the serum natriuretic peptides blood test has been reported, GPs would refer patients for the CORS test who demonstrated elevated levels of natriuretic peptides.^1^ This has the potential to overcome the reduced specificity of the blood test^11,17,18^ and improve referral accuracy. The purpose of this present study was: (i) to determine if patients suspected of HF were able to complete the CORS test after being referred to secondary care and (ii) to evaluate differences in the CORS test parameters between HF and non-HF, and heart failure reduced (HFrEF) vs heart failure preserved (HFpEF) ejection fraction.

## Methods

### Study design, setting, and patients

In this single centre, prospective, observational, feasibility study, 105 consecutive suspected HF patients were recruited from HF diagnostic clinics at the Royal Victoria Infirmary, Newcastle upon Tyne Hospitals National Health Service Foundation Trust, Newcastle upon Tyne, United Kingdom. Data were collected between February 2017 and March 2020. Patients aged ≥50 years were included in the study if they were suspected of having HF by their GPs (based on symptoms, signs, physical examination, and raised natriuretic peptides levels) and had been referred to secondary care to a HF diagnostic clinic for specialist review. Signs and symptoms included breathlessness, ankle swelling, and fatigue, which are found in the ESC HF guidelines.^1^ Exclusion criteria included a previous diagnosis of HF, the presence of severe symptoms requiring urgent assessment and stabilization, recent acute coronary syndrome and severe physical disability. The study protocol (number 16/NE/0287) was approved by the National Health Service, National Research Authority (North East—Tyne & Wear South Research Ethics Committee). All procedures performed in the study were in accordance with the Declaration of Helsinki. Patients gave written informed consent.

### Study protocol and measurements

The CORS test has been described previously.^26^ It uses continuous haemodynamic measurements based on noninvasive, electrical signal processing bioreactance technology (NICOM, Cheetah Medical, Inc., MA, USA), which we have previously validated.^28–30^ The CORS test has demonstrated acceptable reproducibility in healthy adults (≥50 years old)^26^ and consists of 4 phases: (i) rest (patients lies in the supine position for 3 min); (ii) challenge (patients remains in the standing position for 3 min); (iii) and (iv) stress-step exercise (patient completes a step test on a 15-inch step board with intensity controlled by a metronome at 10 and 15 steps per min).^26^ Due to time restrictions in the clinic, patients in this study only completed 1 stress-step exercise phase at the 15 steps per min intensity.

Suspected HF patient NTproBNP (ng/L) levels were recorded from patient records, which the consultant cardiologist would extract as part of the diagnostic tests to confirm or refute a diagnosis of HF. NICE guidelines recommend referral to specialist assessment and echocardiography when NTproBNP levels are between 400 and 2,000 ng/L within 6 weeks whereas levels >2,000 ng/L require referral within 2 weeks.^2^ Patients completed a number of diagnostic tests including echocardiography (estimated left ventricular ejection fraction, LVEF %) and electrocardiography, and were seen by a consultant cardiologist—HF specialist who reviewed their medical history, diagnostic test results, and conducted a physical examination to confirm or rule out a diagnosis of HF. The consultant cardiologist confirmed a diagnosis of HFrEF by estimated LVEF <40% or HFpEF by estimated LVEF >50%. There were 6 patients diagnosed with HF with midrange ejection (HFmrEF) who were grouped with HFrEF for the purposes of this study. All CORS test assessments for this study were administered by a researcher and completed after the patient had been seen by the consultant cardiologist and a diagnosis of HF was confirmed or refuted. The researcher was blinded from echocardiology and patient diagnosis at the time of conducting the CORS test.

### Statistical analysis

Data were analysed using SPSS, version 25 (SPSS, Inc., Chicago, IL, USA). The level of significance was set at *P* <0.05. Data are expressed as mean ± SD unless otherwise indicated. Prior to statistical analysis, data were screened for univariate outliers using standard *Z*-distribution cut-offs. Shapiro–Wilk test was used to assess normality of distribution and Levene’s test confirmed homogeneity of variances. Differences between the 2 groups (confirmed HF and non-HF patients) were assessed using either an independent *t*-test or Mann–Whitney *U* test. In a separate subset analysis, confirmed HF patients only were grouped by ejection fraction diagnosis (HFrEF vs HFpEF) and differences were assessed using either an independent *t*-test or Mann–Whitney *U* test.

## Results

Patients’ demographics, clinical characteristics, and medication list are presented in Table 1. Confirmed HF patients had a significantly lower estimated LVEF % (43 ± 12% vs 54 ± 4%, *P* < 0.01) and higher NTproBNP (1,883 ± 1,957 vs 748 ± 910 ng/L, *P* < 0.01) than non-HF patients. There were no other significant differences between the groups in their demographic and clinical characteristics. All patients completed the rest phase of the CORS test (38 with HF and 67 without HF). While all confirmed HF patients were able to complete the challenge phase, this was the case for 93% of non-HF cases. A total of 79% of newly diagnosed HF patients completed all 3 phases of the CORS test, including the stress-step exercise, whereas 91% of non-HF patients completed all 3 phases of the CORS test. The reasons for missing measurements varied. The consultant cardiologist requested that the patient not complete either the challenge-standing (non-HF patients, *n* = 1) and/or the stress-step exercise (confirmed HF patients, *n* = 4; non-HF patients, *n* = 2) phases due to their condition or there was poor connectivity and signal loss during measurements (challenge-standing phase: non-HF patients, *n* = 4; stress-step exercise phase: confirmed HF patients, *n* = 2; non-HF patients, *n* = 3) or the patient reported balance issues for the stress-step exercise phase and researcher did not complete this phase (confirmed HF patients, *n* = 2; non-HF patients, *n* = 1). For the challenge-standing and stress-step exercise phases that included complete patient haemodynamic measurements but resulted in missing blood pressure measurements were due to poor connectivity or system time out from the bioreactance technology as the patient was not seated at the time of these phase measurements (missing blood pressure measurements: 5% for challenge-standing phase for confirmed HF patients, *n* = 2/38; 23% for stress-step exercise phase for both confirmed HF patients, *n* = 7/30 and non-HF patients, *n* = 14/61).

**Table 1.** Patient demographics, cardiovascular comorbidities, and medications (February 2017–March 2020).

	Confirmed HF	Non-HF	*P*
	*N* = 38	*N* = 67	
Age (years)	73 ± 12	71 ± 9	0.29
Gender, male/female	26/12	31/36	
Height (cm)	170 ± 7	168 ± 10	0.26
Body weight (kg)	89 ± 21	87 ± 22	0.55
Body mass index (kg/m^2^)	31 ± 6	31 ± 7	0.89
LVEF (%)	43 ± 12	54 ± 4	0.00
NTproBNP (ng/L)	1,883 ± 1,957	748 ± 910	0.00
Cardiovascular comorbidities (%)^a^			
Atrial fibrillation	47 (18, 29)	24	
Ischaemic heart disease	21 (11, 11)	9	
Dilated cardiomyopathy	8 (8, 0)	0	
Coronary heart disease	16 (11, 5)	9	
Coronary artery bypass graft	3 (3, 0)	0	
Myocardial infarction	16 (13, 3)	4	
Hypertension	8 (3, 5)	1	
Atrial dysrhythmia	3 (0, 3)	0	
Pulmonary hypertension	3 (0, 3)	1	
Medications (%)^a^			
Angiotensin-converting enzyme inhibitors	53 (32, 21)	33	
Angiotensin II receptor antagonist	24 (11, 13)	16	
Beta-adrenergic blocker	63 (37, 26)	46	
Calcium channel blocker	16 (3, 13)	7	
Diuretics	71 (39, 32)	30	
Anti-arrhythmia	42 (13, 29)	37	
Anti-anginal	3 (0, 3)	9	
Diabetes	13 (11, 3)	21	
Corticosteroids	0 (0, 0)	7	
Anti-inflammatory	0 (0, 0)	10	
Antidepressant	24 (16, 8)	27	
Statins	66 (32, 34)	66	
Anticoagulants	47 (24, 24)	33	

Data are expressed as mean ± SD.

% data are reported in brackets for HFrEF and HFpEF patients.

Table 2 displays the haemodynamic variables for patients with confirmed HF and non-HF for the 3 phases of the CORS test (rest, challenge, and stress-step exercise). At rest, patients with confirmed HF had a significantly higher heart rate (74 ± 16 vs 68 ± 16 beats/min, *P* = 0.03), lower stroke volume (84 ± 28 vs 97 ± 33 mL/beat, *P* = 0.04), lower stroke volume index (SVI; 43 ± 15 vs 51 ± 16 mL/beat/m^2^, *P* = 0.02), and higher diastolic blood pressure (85 ± 12 vs 80 ± 11 mm Hg, *P* = 0.02) than non-HF patients.

**Table 2.** Haemodynamic measures for confirmed and non-HF patients for all phases of the CORS test (February 2017–March 2020).

	Confirmed HF patients	*N*	Non-HF patients	*N*	% difference	*P*
Phase 1—rest						
QT (L/min)	6.0 ± 1.5	38	6.3 ± 1.8	67	5	0.37
CI (L/min/m^2^)	3.0 ± 0.8	38	3.3 ± 0.8	67	10	0.15
HR (beats/min)	74 ± 16	38	68 ± 16	67	8	0.03
SV (mL/beat)	84 ± 28	38	97 ± 33	67	14	0.04
SVI (mL/beat/m^2^)	43 ± 15	38	51 ± 16	67	17	0.02
SBP (mm Hg)	143 ± 25	38	143 ± 21	67	0	0.84
DBP (mm Hg)	85 ± 12	38	80 ± 11	67	6	0.02
MAP (mm Hg)	104 ± 13	38	101 ± 12	67	3	0.25
Phase 2—challenge						
QT (L/min)	5.7 ± 1.3	38	5.6 ± 1.6	62	2	0.43
CI (L/min/m^2^)	2.9 ± 0.7	38	2.9 ± 0.7	62	0	0.8
HR (beats/min)	81 ± 18	38	72 ± 14	62	12	0.01
SV (mL/beat)	73 ± 21	38	79 ± 24	62	8	0.35
SVI (mL/beat/m^2^)	38 ± 11	38	41 ± 11	62	8	0.18
SBP (mm Hg)	138 ± 22	36	144 ± 22	62	4	0.19
DBP (mm Hg)	83 ± 11	36	83 ± 12	62	0	0.74
MAP (mm Hg)	102 ± 12	36	104 ± 14	62	2	0.44
Phase 3—stress-step exercise						
QT (L/min)	9.4 ± 3.3	30	10.4 ± 3.7	61	10	0.23
CI (L/min/m^2^)	4.8 ± 1.5	30	5.5 ± 1.8	61	14	0.12
HR (beats/min)	100 ± 24	30	93 ± 16	61	7	0.36
SV (mL/beat)	96 ± 32	30	113 ± 37	61	16	0.04
SVI (mL/beat/m^2^)	49 ± 16	30	58 ± 17	61	17	0.02
SBP (mm Hg)	150 ± 30	23	150 ± 26	47	0	0.93
DBP (mm Hg)	91 ± 13	23	92 ± 17	47	1	0.71
MAP (mm Hg)	110 ± 18	23	112 ± 18	47	2	0.69

CI, cardiac index; DBP, diastolic blood pressure; HR, heart rate; MAP, mean arterial pressure; QT, cardiac output; SBP, systolic blood pressure; SV, stroke volume. Data are expressed as mean ± SD.

For the challenge phase of the CORS test, heart rate was significantly higher in the confirmed HF patients (81 ± 18 vs 72 ± 14 beats/min, *P* = 0.01) but no other variables were significantly different between confirmed HF and non-HF. Patients with confirmed HF had a significantly lower SVI compared with the non-HF group for the stress-step exercise phase of the CORS test (49 ± 16 vs 58 ± 17, mL/beat/m^2^, *P* = 0.02).

In the subset analysis of confirmed HF patients only, there were 21 HFrEF (males, *n* = 15, females, *n* = 6; LVEF = 35 ± 10%) patients and 17 (males, *n* = 11, females, *n* = 6; LVEF = 53 ± 3%) HFpEF patients. Patients with HFpEF were significantly older than patients with HFrEF (81 ± 7 vs 68 ± 11 years old, *P* < 0.01) No significant differences were found between patients with HFrEF and HFpEF for body mass index (31 ± 7 vs 30 ± 5 kg/m^2^, *P* = 0.63) and NTproBNP (1,851 ± 2,547 vs 1,909 ± 1,478 ng/L, *P* = 0.95). Table 3 shows the haemodynamic variables for both HFrEF and HFpEF patients for the 3 phases of the CORS test (rest, challenge, and stress-step exercise). At all 3 phases of the CORS test, patients with HFrEF had significantly higher heart rate (80 ± 18 vs 67 ± 11 beats/min, *P* = 0.02; 88 ± 20 vs 73 ± 12 beats/min, *P* = 0.01; and 106 ± 22 vs 91 ± 24 beats/min, *P* = 0.04) compared with patients with HFpEF. SVI was significantly lower in patients with HFrEF at the rest and challenge phases (39 ± 15 vs 48 ± 13 mL/beat/m^2^, *P* = 0.02 and 34 ± 9 vs 42 ± 12 mL/beat/m^2^, *P* = 0.03, respectively) than patients with HFpEF. Systolic blood pressure was lower in patients with HFrEF at the challenge phase compared with patients with HFpEF (132 ± 20 vs 147 ± 22 mm Hg, *P =* 0.05). No other significant differences were found between the 2 groups.

**Table 3.** Haemodynamic measures for confirmed HF patients for all phases of the CORS test (February 2017–March 2020).

	HFrEF	*N*	HFpEF	*N*	% difference	*P*
Phase 1—rest						
QT (L/min)	6.0 ± 1.6	21	6.0 ± 1.3	17	0	0.92
CI (L/min/m^2^)	3.0 ± 0.8	21	3.1 ± 0.7	17	3	0.57
HR (beats/min)	80 ± 18	21	67 ± 11	17	18	0.02
SV (mL/beat)	78 ± 27	21	92 ± 29	17	16	0.15
SVI (mL/beat/m^2^)	39 ± 15	21	48 ± 13	17	21	0.02
SBP (mm Hg)	135 ± 23	21	151 ± 25	17	11	0.09
DBP (mm Hg)	87 ± 11	21	82 ± 12	17	6	0.23
MAP (mm Hg)	103 ± 14	21	105 ± 13	17	2	0.69
Phase 2—challenge						
QT (L/min)	5.6 ± 1.3	21	5.7 ± 1.4	17	2	0.67
CI (L/min/m^2^)	2.8 ± 0.7	21	3.0 ± 0.8	17	7	0.44
HR (beats/min)	88 ± 20	21	73 ± 12	17	19	0.01
SV (mL/beat)	67 ± 16	21	81 ± 24	17	19	0.05
SVI (mL/beat/m^2^)	34 ± 9	21	42 ± 12	17	21	0.03
SBP (mm Hg)	132 ± 20	21	147 ± 22	15	11	0.05
DBP (mm Hg)	85 ± 12	21	81 ± 11	15	5	0.37
MAP (mm Hg)	101 ± 13	21	103 ± 11	15	2	0.57
Phase 3—stress-step exercise						
QT (L/min)	9.5 ± 3.5	17	9.2 ± 3.3	13	3	0.71
CI (L/min/m^2^)	4.7 ± 1.5	17	4.8 ± 1.6	13	2	0.93
HR (beats/min)	106 ± 22	17	91 ± 24	13	15	0.04
SV (mL/beat)	90 ± 31	17	103 ± 33	13	13	0.28
SVI (mL/beat/m^2^)	46 ± 16	17	54 ± 16	13	16	0.18
SBP (mm Hg)	141 ± 27	13	162 ± 29	10	14	0.09
DBP (mm Hg)	88 ± 12	13	93 ± 15	10	6	0.38
MAP (mm Hg)	105 ± 17	13	116 ± 17	10	10	0.14

CI, cardiac index; DBP, diastolic blood pressure; HR, heart rate; MAP, mean arterial pressure; QT, cardiac output; SBP, systolic blood pressure; SV, stroke volume. Data are expressed as mean ± SD.

## Discussion

The purpose of the present study was to determine the feasibility for completing the CORS test in suspected HF patients and to evaluate the differences in the CORS test parameters between HF and non-HF and patients with HFrEF and HFpEF. The preliminary findings highlight that nearly 80% of newly diagnosed HF patients were able to successfully complete all phases of the CORS test (including step exercise). There were differences in haemodynamic function between confirmed HF and non-HF patients at each of the phases of the CORS test. There were further differences between patients with HFrEF and those with a diagnosis of HFpEF.

The ESC HF guidelines defines HF as a clinical syndrome associated with cardiac functional and/or structural abnormalities resulting in reduced cardiac output at rest and/or during stress.^1^ However, in the description of diagnostic pathway there is no indication about monitoring and reporting haemodynamic measurements in patients undergoing HF diagnostic clinical investigation. Thus, we have developed the CORS test to evaluate whether monitoring haemodynamics in patients with suspected HF at rest and in response to stress, using a simple and noninvasive technology, may help primary care physicians to more accurately rule-in or rule-out HF in conjunction with other accessible investigations including medical history, physical examination, electrocardiogram, and natriuretic peptide test. Primary care is recognized as a key area to develop new strategies to achieve timely HF diagnosis and in this study the CORS test has demonstrated some important findings in newly diagnosed HF patients.^25^

The present study reveals that the majority of patients were able to complete all phases of the CORS test (rest-supine, challenge-standing, and stress-step exercise). This was a concern raised by healthcare professionals during our qualitative evaluation of the potential implementation of the CORS test for suspected HF in primary care.^27^ The CORS test was administered in 18 ± 3 min so an additional appointment will be required for this test to be included within primary care. The qualitative work highlighted that not all phases of the test would be suitable for all patients.^27^ This current study has seen adaptations to the CORS test such as reducing the 2-phase stress-step exercise (originally proposed^26^) to 1 phase to be time efficient and using a balance aid for the stress-step exercise phase when the patient required, which are important developments if as suggested from our qualitative work that either a practice nurse or healthcare assistant would be able to deliver the test within a primary care setting.^27^ Using such approach we achieved a 93% and 79% success rate for completion of the stress-exercise CORS test in patients with suspected HF and those with confirmed HF. This finding addresses previous concerns identified in our qualitative work by GPs and practice nurses that completion of the CORS test will be achieved in only 50%–80% of patients.^27^ It should also be noted that haemodynamic measurements during the rest phase of the CORS test could be achieved in all patients, and can yield important information about the presence and type of HF, as described below.

In patients with confirmed HF, heart rate was significantly higher at both rest and challenge phases compared with non-HF patients. Likewise, for all 3 phases of the CORS test, heart rate was higher in patients with HFrEF compared with patients with HFpEF. In patients with HFrEF, findings highlight that, for every beat increase in heart rate, there is a 3% increase in risk of mortality or hospital admission for worsening HF.^31^ Heart rate control is a vital part of HF management.^31,32^ As the patients in our study were recruited from a diagnostic clinic, this was before treatment optimization.

Both the rest and stress-step exercise phases resulted in a lower stroke volume and/or SVI for confirmed HF patients compared with non-HF patients. The differences in the subset analysis of confirmed HF patients revealed differences in SVI at the rest and challenge phases of the CORS test with lower readings for patients with HFrEF vs patients with HFpEF. Lower stroke volume has been reported previously in patients with HFrEF at rest and at exercise compared with patients with HFpEF.^33^ Similarly, lower SVI has been found at rest in patients with HFrEF (*n* = 157) compared with healthy participants (*n* = 147) using cardiovascular magnetic resonance.^34^

Diastolic blood pressure was significantly different at rest with a higher reading recorded in the confirmed HF group (vs non-HF group). No other significant blood pressure differences were recorded between groups. Although, in the subset analysis, systolic blood pressure was lower in the HFrEF group at the challenge phase compared with the HFpEF group. Interestingly, in the confirmed HF group, systolic and diastolic blood pressure decreased from supine to challenge phases. Abnormal haemodynamic postural response differences have been found previously in HF patients with lower blood pressure recorded at a standing position vs supine, which may present as postural hypotension.^35^

For the first time, we have provided results from suspected HF patients completing the CORS test. However, this study is not without limitations. This was a single centre, prospective observational, feasibility study with moderate sample size. Our ongoing research programme aims to confirm the diagnostic accuracy (sensitivity and specificity) of the CORS test with the objective of its implementation in primary care to improve diagnosis of HF. Although at the time of performing the CORS test, all patients were suspected of having HF, Table 1 shows differences in medication used at the time of investigations, which may influence haemodynamic function. We acknowledge that at the point of HF diagnosis that medication was not titrated. We did not use a visual analogue scale to assess patient burden of completing the CORS test, which would have strengthened our feasibility findings. The patient was not blinded from their diagnosis at the time of completing the CORS test and we acknowledge that this may have impacted on their well-being and capacity to perform the test. The patients were seen by one of 3 consultant cardiologists (HF specialists), however, we did not complete inter-rater variability between the clinicians regarding HF diagnosis.

The major findings of the present study highlight that the CORS test can be completed by the majority of patients undergoing a clinical investigation for a diagnosis of HF within a secondary care setting. The findings have shown that performing the CORS test in HF suspected patients is feasible within a secondary care setting. Furthermore study results demonstrate haemodynamic differences between HF and non-HF as well as HFrEF vs HFpEF. It appears that SVI at rest and its response to challenge (standing) and/or short step exercise differentiate the most between patients with HF from non-HF patients and HFrEF from HFpEF. Our ongoing research will define cut-off values for the CORS test variables and confirm their sensitivity and specificity to complement diagnosis of HF in primary care.

## Supplementary Material

cmab184_suppl_Supplementary_Checklist

## Data Availability

The data underlying this article will be shared on reasonable request to the corresponding author.

## References

[1] Ponikowski P , Voors AA, Anker SD, Bueno H, Cleland JG, Coats AJ, Falk V, Gonzalez-Juanatey JR, Harjola VP, Jankowska EA, et al. 2016 ESC Guidelines for the diagnosis and treatment of acute and chronic heart failure: the Task Force for the diagnosis and treatment of acute and chronic heart failure of the European Society of Cardiology (ESC) Developed with the special contribution of the Heart Failure Association (HFA) of the ESC. Eur Heart J. 2016;37(27):2129–2200. 27206819 10.1093/eurheartj/ehw128

[2] National Institute for Health and Care Excellence. Chronic heart failure in adults: diagnosis and management. 2018. 30645061

[3] Ambrosy AP , Fonarow GC, Butler J, Chioncel O, Greene SJ, Vaduganathan M, Nodari S, Lam CSP, Sato N, Shah AN, et al. The global health and economic burden of hospitalizations for heart failure: lessons learned from hospitalized heart failure registries. J Am Coll Cardiol. 2014;63(12):1123–1133. 24491689 10.1016/j.jacc.2013.11.053

[4] Conrad N , Judge A, Canoy D, Tran J, Pinho-Gomes AC, Millett ERC, Salimi-Khorshidi G, Cleland JG, McMurray JJV, Rahimi K. Temporal trends and patterns in mortality after incident heart failure: a longitudinal analysis of 86 000 individuals. JAMA Cardiol. 2019;4(11):1102–1111. 31479100 10.1001/jamacardio.2019.3593 PMC6724155

[5] Conrad N , Judge A, Tran J, Mohseni H, Hedgecott D, Crespillo AP, Allison M, Hemingway H, Cleland JG, McMurray JJ. Temporal trends and patterns in heart failure incidence: a population-based study of 4 million individuals. Lancet. 2018;391(10120):572–580. 29174292 10.1016/S0140-6736(17)32520-5 PMC5814791

[6] Taylor CJ , Ryan R, Nichols L, Gale N, Hobbs FR, Marshall T. Survival following a diagnosis of heart failure in primary care. Fam Pract. 2017;34(2):161–168. 28137979 10.1093/fampra/cmw145 PMC6192063

[7] Hobbs FD , Roalfe AK, Davis RC, Davies MK, Hare R. Prognosis of all-cause heart failure and borderline left ventricular systolic dysfunction: 5 year mortality follow-up of the Echocardiographic Heart of England Screening Study (ECHOES). Eur Heart J. 2007;28(9):1128–1134. 17459902 10.1093/eurheartj/ehm102

[8] National Institute for Health and Clinical Excellence. Chronic heart failure: national clinical guideline for diagnosis and management in primary and secondary care. 2010.

[9] Mant J , Doust J, Roalfe A, Barton P, Cowie MR, Glasziou P, Mant D, McManus RJ, Holder R, Deeks J, et al. Systematic review and individual patient data meta-analysis of diagnosis of heart failure, with modelling of implications of different diagnostic strategies in primary care. Health Technol Assess. 2009;13(32):1–207. 10.3310/hta13320 19586584

[10] Taylor CJ. Diagnosing heart failure: challenges in primary care. Heart. 2019;105(9):663–664. 30647095 10.1136/heartjnl-2018-314396

[11] Hobbs FD , Doust J, Mant J, Cowie MR. Heart failure: diagnosis of heart failure in primary care. Heart. 2010;96(21):1773–1777. 20956495 10.1136/hrt.2007.139402

[12] British Society for Heart Failure. National Heart Failure Audit 2016–2017 summary report. 2018.

[13] Taylor CJ , Rutten FH, Brouwer JR, Hobbs FR. Practical guidance on heart failure diagnosis and management in primary care: recent EPCCS recommendations. Br J Gen Pract. 2017;67(660):326–327. 28663432 10.3399/bjgp17X691553 PMC5565867

[14] Roalfe AK , Mant J, Doust JA, Barton P, Cowie MR, Glasziou P, Mant D, McManus RJ, Holder R, Deeks JJ, et al. Development and initial validation of a simple clinical decision tool to predict the presence of heart failure in primary care: the MICE (Male, Infarction, Crepitations, Edema) rule. Eur J Heart Fail. 2012;14(9):1000–1008. 22713289 10.1093/eurjhf/hfs089

[15] Khunti K , Squire I, Abrams KR, Sutton AJ. Accuracy of a 12-lead electrocardiogram in screening patients with suspected heart failure for open access echocardiography: a systematic review and meta-analysis. Eur J Heart Fail. 2004;6(5):571–576. 15352303 10.1016/j.ejheart.2004.03.013

[16] Doust JA , Glasziou PP, Pietrzak E, Dobson AJ. A systematic review of the diagnostic accuracy of natriuretic peptides for heart failure. Arch Intern Med. 2004;164(18):1978–1984. 15477431 10.1001/archinte.164.18.1978

[17] Roberts E , Ludman AJ, Dworzynski K, Al-Mohammad A, Cowie MR, McMurray JJ, Mant J. The diagnostic accuracy of the natriuretic peptides in heart failure: systematic review and diagnostic meta-analysis in the acute care setting. BMJ. 2015;350:h910. 25740799 10.1136/bmj.h910 PMC4353288

[18] Taylor CJ , Roalfe AK, Iles R, Hobbs FR, Barton P, Deeks J, McCahon D, Cowie MR, Sutton G, Davis RC, et al. Primary care REFerral for EchocaRdiogram (REFER) in heart failure: a diagnostic accuracy study. Br J Gen Pract. 2017;67(655):e94–e102. 27919937 10.3399/bjgp16X688393 PMC5308123

[19] Valk M , Hoes AW, Mosterd A, Broekhuizen B, Zuithoff N, Rutten FH. Time trends in the use and appropriateness of natriuretic peptide testing in primary care: an observational study. BJGP Open. 2020;4(4):bjgpopen20X101074. 10.3399/bjgpopen20X101074 PMC7606146 32788172

[20] Kelder JC , Cramer MJ, van Wijngaarden J, van Tooren R, Mosterd A, Moons KG, Lammers JW, Cowie MR, Grobbee DE, Hoes AW. The diagnostic value of physical examination and additional testing in primary care patients with suspected heart failure. Circulation. 2011;124(25):2865–2873. 22104551 10.1161/CIRCULATIONAHA.111.019216

[21] Fonseca C. Diagnosis of heart failure in primary care. Heart Fail Rev. 2006;11(2):95–107. 16937029 10.1007/s10741-006-9481-0

[22] Fuat A , Hungin AP, Murphy JJ. Barriers to accurate diagnosis and effective management of heart failure in primary care: qualitative study. BMJ. 2003;326(7382):196. 12543836 10.1136/bmj.326.7382.196 PMC140276

[23] Moayedi Y , Alba AC, Lee DS, Wijeysundera HC, Ross HJ. The next wave of health care strain related to COVID-19: heart failure patients coming back in force: we must not fail them. Can J Cardiol. 2020;36(7):993–994. 32504660 10.1016/j.cjca.2020.05.037 PMC7834641

[24] George I , Salna M, Kobsa S, Deroo S, Kriegel J, Blitzer D, Shea NJ, D\'Angelo A, Raza T, Kurlansky P, et al. The rapid transformation of cardiac surgery practice in the coronavirus disease 2019 (COVID-19) pandemic: insights and clinical strategies from a centre at the epicentre. Eur J Cardiothorac Surg. 2020;58(4):667–675. 32573737 10.1093/ejcts/ezaa228 PMC7337744

[25] Taylor CJ , Ordóñez-Mena JM, Roalfe AK, Lay-Flurrie S, Jones NR, Marshall T, Hobbs FDR. Trends in survival after a diagnosis of heart failure in the United Kingdom 2000–2017: population based cohort study. BMJ. 2019;364:l223. 30760447 10.1136/bmj.l223 PMC6372921

[26] Charman SJ , Okwose NC, Stefanetti RJ, Bailey K, Skinner J, Ristic A, Seferovic PM, Scott M, Turley S, Fuat A, et al. A novel cardiac output response to stress test developed to improve diagnosis and monitoring of heart failure in primary care. ESC Heart Fail. 2018;5(4):703–712. 29943902 10.1002/ehf2.12302 PMC6073030

[27] Charman S , Okwose N, Maniatopoulos G, Graziadio S, Metzler T, Banks H, Vale L, MacGowan GA, Seferović PM, Fuat A, et al. Opportunities and challenges of a novel cardiac output response to stress (CORS) test to enhance diagnosis of heart failure in primary care: qualitative study. BMJ Open. 2019;9(4):e028122. 10.1136/bmjopen-2018-028122 PMC6500186 30987993

[28] Perkins RE , Hollingsworth KG, Eggett C, MacGowan GA, Bates MG, Trenell MI, Jakovljevic DG. Relationship between bioreactance and magnetic resonance imaging stroke volumes. Br J Anaesth. 2016;117(1):134–136. 27317716 10.1093/bja/aew164 PMC4913415

[29] Jones TW , Houghton D, Cassidy S, MacGowan GA, Trenell MI, Jakovljevic DG. Bioreactance is a reliable method for estimating cardiac output at rest and during exercise. Br J Anaesth. 2015;115(3):386–391. 25659999 10.1093/bja/aeu560

[30] Jakovljevic DG , Trenell MI, MacGowan GA. Bioimpedance and bioreactance methods for monitoring cardiac output. Best Pract Res Clin Anaesthesiol. 2014;28(4):381–394. 25480768 10.1016/j.bpa.2014.09.003

[31] Böhm M , Swedberg K, Komajda M, Borer JS, Ford I, Dubost-Brama A, Lerebours G, Tavazzi L. Heart rate as a risk factor in chronic heart failure (SHIFT): the association between heart rate and outcomes in a randomised placebo-controlled trial. Lancet. 2010;376(9744):886–894. 20801495 10.1016/S0140-6736(10)61259-7

[32] Fu M , Ahrenmark U, Berglund S, Lindholm CJ, Lehto A, Broberg AM, Tasevska-Dinevska G, Wikstrom G, Ågard A, Andersson B, et al. Adherence to optimal heart rate control in heart failure with reduced ejection fraction: insight from a survey of heart rate in heart failure in Sweden (HR-HF study). Clin Res Cardiol. 2017;106(12):960–973. 28795299 10.1007/s00392-017-1146-6 PMC5696492

[33] Tsujinaga S , Iwano H, Chiba Y, Ishizaka S, Sarashina M, Murayama M, Nakabachi M, Nishino H, Yokoyama S, Okada K, et al. Heart failure with preserved ejection fraction vs. reduced ejection fraction—mechanisms of ventilatory inefficiency during exercise in heart failure. Circ Rep. 2020;2(5):271–279. 33693241 10.1253/circrep.CR-20-0021 PMC7925313

[34] Carlsson M , Andersson R, Bloch KM, Steding-Ehrenborg K, Mosén H, Stahlberg F, Ekmehag B, Arheden H. Cardiac output and cardiac index measured with cardiovascular magnetic resonance in healthy subjects, elite athletes and patients with congestive heart failure. J Cardiovasc Magn Reson. 2012;14(1):51. 22839436 10.1186/1532-429X-14-51 PMC3419124

[35] Bronzwaer, T, Bogert LWJ, Westerhof BE, Piek JJ, Daemen MJAP, van Lieshout JJ. Abnormal haemodynamic postural response in patients with chronic heart failure. ESC Heart Fail. 2017;4(2):146–153. 28451451 10.1002/ehf2.12127 PMC5396043

